# Culturally responsive approaches to cultivate care and innovation among emerging public health leaders for ethical community engagement: perspectives informed through lived experience

**DOI:** 10.3389/fpubh.2025.1602187

**Published:** 2025-06-18

**Authors:** Marina Suzanne Hernandez, Ruth Murcia

**Affiliations:** Department of Africana, Latin American, Caribbean, & Latinx Studies, University at Albany, Albany, NY, United States

**Keywords:** innovation, health leadership, research methods, community engagement, participatory methods, pedagogy, Latinx studies, humanities and social science

## Abstract

Traditional methods of public health research, practice, and education continue to overlook the value of multidisciplinary approaches to research, practice, and training in addressing health problems. Students who graduate from public health programs gain insufficient exposure to other fields of study and lack the leadership skills to effectively navigate interprofessional teams. Generally, public health programs do not adequately prepare students to engage with scholars from other fields such as humanities, ethnic studies, gender studies, etc. whose dynamic perspectives have not traditionally been considered in public health frameworks. Students, thus, become professionals who are ill-equipped to apply transdisciplinary approaches that critically examine the complex landscape of social health determinants and evolving health crises. Moreover, emerging student leaders with intimate connections to communities of interest are forced to shed their identities to conform to public health “best practices.” We aim to strengthen leadership development in public health programs through innovative research methods and collaborative pedagogies. We critique the conceptualization of “interdisciplinarity” within the public health field, demonstrate the potential of innovative methods to responsibly engage with culturally diverse communities, and propose strategies to strengthen community-researcher collaboration to foster more robust leadership skills among public health scholars. Our recommendations integrate diverse tools and resources from other fields of study that will achieve more equitable health solutions.

## Introduction

Given the landscape of increasing mistrust, and distrust, of public health leadership in the United States, fresh and critical perspectives on public health education and cultural responsiveness are key to rebuilding a relationship on the foundations of care (1, 2). Community participation requires full integration and leadership of community members, not merely an integration of their thoughts, opinions, and labor as data collectors or implementors (3). In this article, we critically examine common practices in public health education and practice to generate actionable steps in strengthening relationships between public health leaders and exceptionally vulnerable populations.

We aim to foster more robust leadership skills among public health professions through responsible engagement with historically excluded and abused communities. As scholars in both public health and Latina/o/x Studies, we bring a fresh perspective on public health leadership and community engagement through our application of community-academic praxis to public health work (4). Our engagement with vulnerable populations, including our own communities, and interdisciplinary training offer an approach to reshaping public health leadership strategies worthy of consideration.

First, we characterize the problem of mistrust and distrust among historically excluded and abused communities. Black, Hispanic/Latinx, and Indigenous people as well as individuals who identify as 2SBGLTQIAP + have an exceptionally troubled history with science and medicine in the United States (5). The problem the public health field is facing with popular trust is less a result of misinformation and rise in “bad science” and more so an issue of historical disempowerment, neglect, and mistreatment of certain communities. We then discuss the two main ingredients (i.e., care and interdisciplinarity) we believe are necessary but missing from public health paradigms to stimulate innovation among emerging health leaders. Our proposed formula suggests that leadership rooted in care fosters trust and sustainable health interventions that meet the unique circumstances of each community of interest. Finally, we conclude with recommended methodologies, pedagogies, and resources for further learning to cultivate care and innovation among both emerging and seasoned public health leaders.

## The problem

The problem of mistrust stems from theories and practices that fail to adequately consider diverse cultural and historical perspectives among certain populations and their needs (2, 6). Public health as a field must accept accountability for our role in generating mistrust so we can begin to rebuild it (2, 6). Scholars agree that health messaging and data are largely designed through frameworks and standards built around able-bodied, White/European descent, and cisgender heterosexual populations (7, 8). Repeated application of hegemonic models creates a wall between public health leaders and vulnerable communities of interest and makes health messages less effective. Culturally dismissive models not only lead to persistent health disparities but also discredit public health agencies because their recommendations fail to work. Feeling betrayed and underserved, historically excluded and abused communities may then be vulnerable to disingenuous sources of information (9, 10). Bad actors exploit people’s desire for visibility and make it more difficult to regain trust (2, 9).

Borderon et al. (7) developed the *Scales of Invisibility* to illustrate how public health curriculum and professional practice have made certain groups of people invisible or misrepresented in data, project design, and research processes. The lack of intentional engagement with certain groups leads to the mischaracterization of marginalized communities as “hard-to-reach” when the issue is more a matter of systematic exclusion (11). Although special methods have been developed to specifically include marginalized communities in public health research such as snowball sampling and respondent-driven sampling, these methods tend to limit community members to data collection without actually extending a leadership role. In our experience, inclusion has prompted more opportunities to be present at the table. We suggest conceptualizing inclusion as having a seat at the table, extending an invitation to be present. Engagement, however, entails decision-making power. Not only should there be diverse voices at the table, but those voices should also be incorporated into decisions (1).

**Figure 1:**
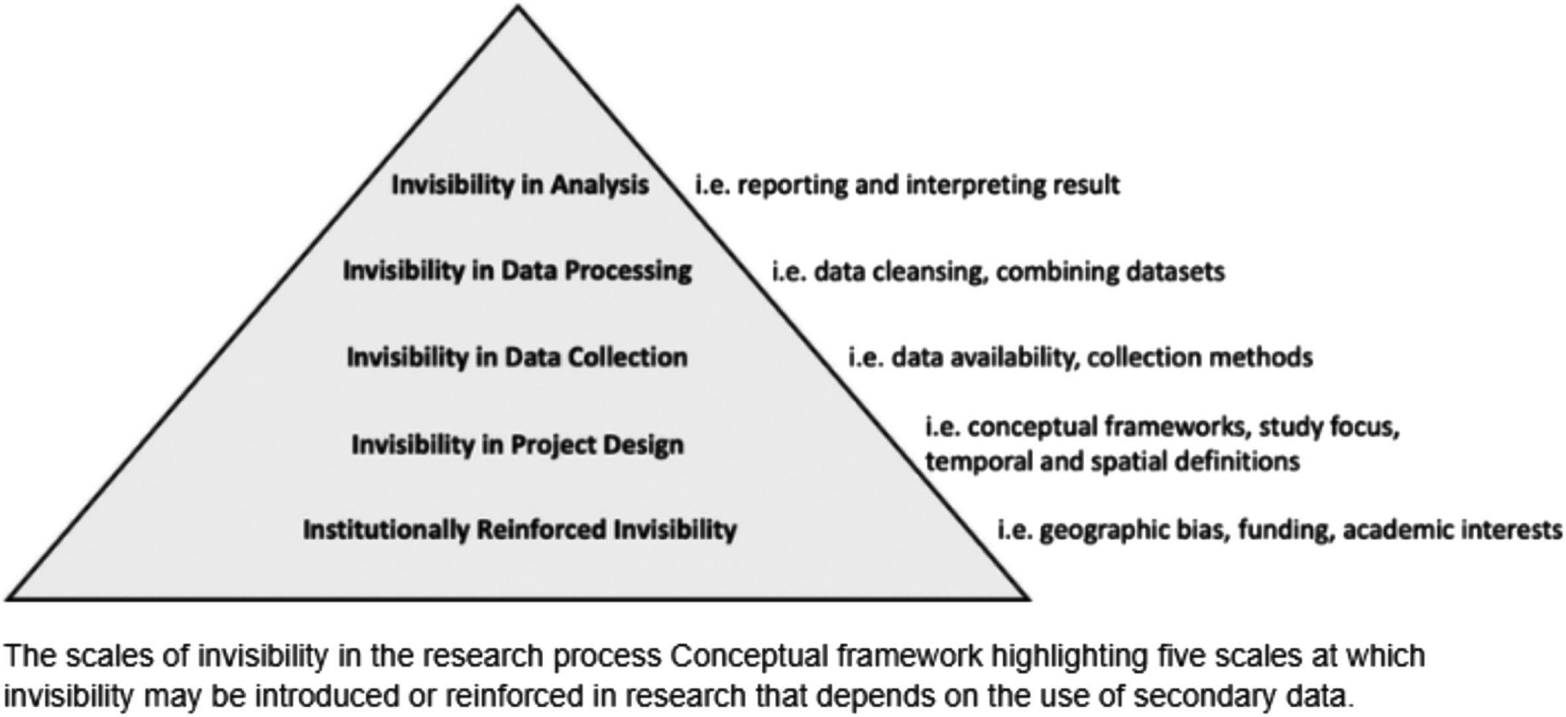
Scales of invisibility (7).

We draw from the *Scales of invisibility* (7) (Figure 1) framework to help public health leaders in research and education disrupt systems that perpetuate a lack of trust. Each of the scales highlights the ways invisibility may be produced in the research process that potentially transfer to public health programs/intervention. We find Borderon et al.’s (7) model relevant to our analysis with emphasis on the first three scales: research focus, project design, and data collection. Scale one *invisibility in research focus*: invites us to reflect the selective ways the discipline or institution allocate funding and favor topics, methods, and questions that neglect some populations and geographies in the scientific investigation. Scale two *Invisibility in project design* assess ways in which invisibility may be introduced during project design through choice of conceptual frameworks, research question and hypothesis formation. We believe that public health frameworks need to be expanded to break the division between researchers and participants, between leaders and communities. Scale three *invisibility in data collection* speaks to types of data and research methods that fail to account for disproportionate representation. Each community has its own way of identifying what data is and how they want to use it to inform interventions, which are likely to be more effective than traditional public health approaches in meeting their collective needs (1).

## Innovation and collaboration in community-public health engagement

Credibility and interdisciplinarity are essential building blocks for responsible engagement with historically excluded and abused communities. As previously discussed, traditional public health frameworks cannot adequately address the unique needs of communities who have been rendered invisible (7, 10). Instead, we need to develop innovative approaches to more effectively address health disparities among vulnerable populations that do not further stigmatize, pathologize, or worsen their circumstances. We recognize there are challenges in pursuing innovative and collaborative strategies in public health work (12). Among the more complicated challenges are logistics of learning new methods, coordinating scheduled activities and cognitive dissonance. Believe one of the most difficult challenges is cognitive dissonance. It is very uncomfortable to be confronted by practices and views that challenge our perceptions of fact, objectivity, and health authority (8, 10). As uncomfortable as it is, cognitive dissonance is crucial to identifying implicit biases so we can address them and shift how we approach our practice (13). This section will discuss the importance of building credibility and challenge the concept of interdisciplinarity.

### (re)Building credibility

Building credibility with learners and research collaborators, revisiting how we practice interdisciplinarity, and utilizing the resources that already exist are crucial first steps in repairing trust (14). Goodwill, or perceived care, is necessary to build credibility with learners and communities. Public health scholars are typically trained to facilitate community *entry* rather than community *care*, which we argue contributes to the problem of widespread lack of trust toward public health actors. For example, students are taught to *enter* a community through gatekeepers without forming a relationship with the community at large (9). We suggest researchers and students make genuine efforts to learn about their communities of interest by first engaging with them outside of a professional capacity. Attending local cultural events, hiring community organizations to conduct workshops with one’s team or institution, and connecting through creative, artistic initiatives from within the community are optimal starting points (15, 16). For example, in Oklahoma, the Hispanic/Latinx community coordinates an annual “OK Cine Latino Film Festival” that features local filmmakers of all ages (17). Not only would attending or volunteering at an event like this expose public health students and professionals to hidden narratives of local Hispanic/Latinx experiences, but it is also an opportunity to eat, drink, learn from, and support the plethora of small Hispanic/Latinx owned businesses in the area (18). Essentially, we believe communities will not show up for us if we do not first show up for them and demonstrate care.

### Rethinking interdisciplinarity

It is unrealistic to expect relationships with communities of interest to flourish without collaborating with other disciplines (12, 19). As we have discussed, hegemonic paradigms in public health are not appropriate for historically excluded and abused communities (10). To cultivate innovation in public health, students must be introduced to different epistemologies from the perspectives of public health (12, 20). We, the authors, boldly claim that public health has not fully gestated as an interdisciplinary field. While we respect and promote current efforts to expand interprofessional engagement, we feel there are structural changes that have yet to be addressed (12, 21).

**Figure 2:**
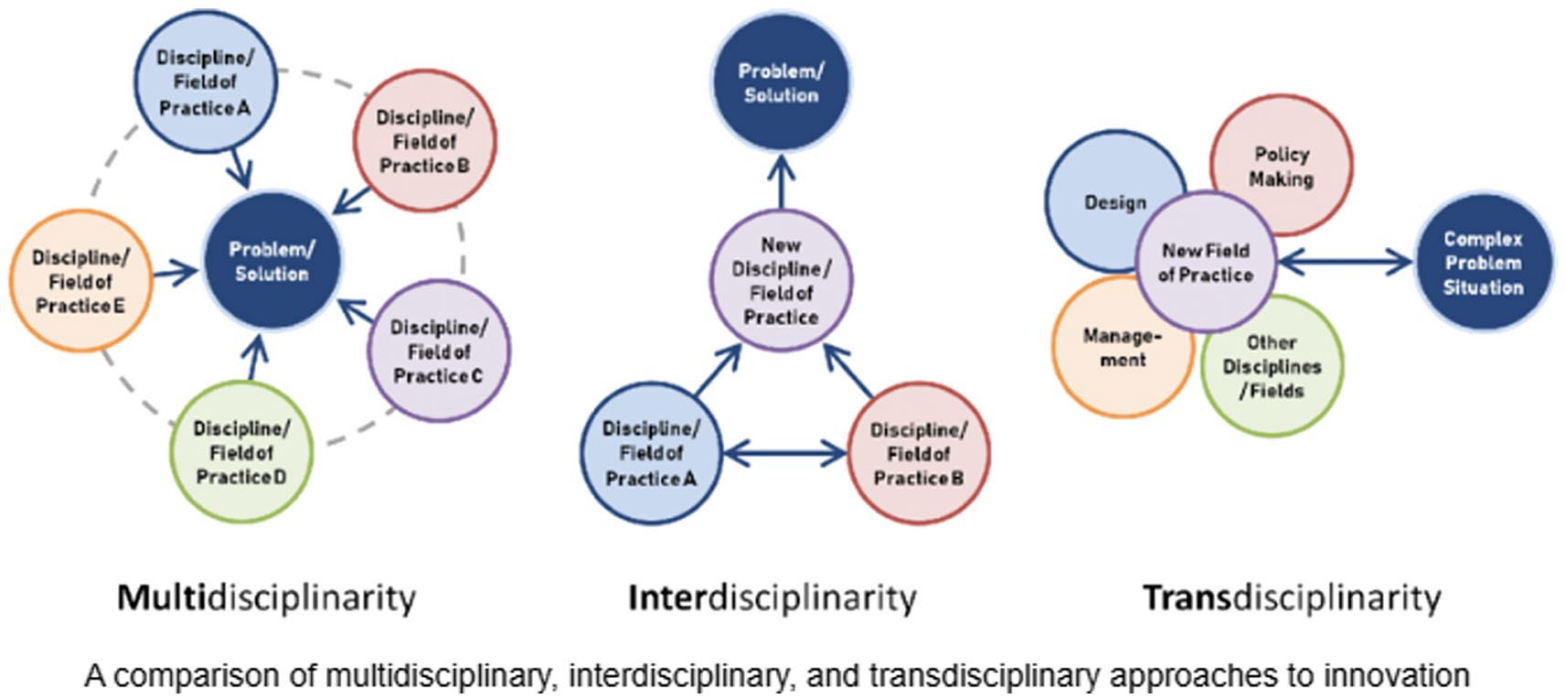
Model of transdisciplinary innovation (22).

McPhee et al. (22) illustrate the characteristics of multi-disciplinarity, interdisciplinarity, and trans-disciplinarity (Figure 2). We support McPhee et al.’s (22) claim that innovation lies in trans-disciplinarity; thus, public health cannot cultivate effective leaders without strategically facilitating exposure to a variety of disciplines, fields of study, and interprofessional engagement starting in the classroom (19, 21). In our experience, public health faculty often refer to the field as interdisciplinary when degree programs require courses in epidemiology, health policy/management, biostatistics, and social/behavioral health. We feel these facets of public health are all rooted in the same core competencies and are ultimately working toward the same interests—identifying and manipulating health outcomes—and/or follow the same models of health promotion; therefore, standard public health education programs do not align with interdisciplinarity much less trans-disciplinarity (19, 22, 43).

Finally, public health leaders need to disrupt colonial practices that perpetuate the myth that science is unbiased (8, 23). Who we are and our social interactions shape our worldviews, which in effect shapes the way we identify health problems and interventions (8). Interprofessional engagement with scholars from Humanities, Ethnic Studies, Gender/Sexuality Studies, Literature Studies, Film Studies, etc. spark a level of consciousness that public health programs are not necessarily equipped to provoke (12, 20, 21).

## Recommendations

We have discussed how perpetuating invisibility in the research process and histories of scientific and medical abuse creates mistrust and distrust. We also discussed the need to build credibility through demonstrations of goodwill and expand the conceptualization of interdisciplinarity in public health. Now, we would like to suggest specific methods and pedagogies that have been developed by scholars with lived experience. We want to emphasize that these methods should not be utilized or implemented without the permission and leadership of the people who own these practices and the scholars with lived experience who have developed these methods.

### Research methodologies

The methodologies suggested below are inspired by recommended practices in African(a) Studies, Latina/o/x Studies, and Indigenous research methods, all of which are regarded as interdisciplinary fields of study (19, 20). Our suggestions are by no means exhaustive but are intended to serve as a starting point to think about culturally responsive means of data collection and community engagement without retraumatizing, exploiting, or reinforcing colonialism in research among vulnerable populations.

Historically excluded and abused communities often form special ways of communicating that, when applied with care and community leadership, can reap valuable insights for public health work. Pláticas and porch-sitting, for example, are intimate spaces for storytelling among Hispanic/Latinx women and Black/African American elders, both of whom have extensive experiences of institutional violence (20, 24). Pláticas methodology is a conversational testimonio that disrupts traditional interviewer-narrator divide by situating participants as co-producers of knowledge (25). Pláticas emphasize everyday lived experiences and offer a more casual, culturally responsive environment than traditional data collection methods. Similarly, the activity of porch-sitting is a cherished ritual among Black/African American elders, primarily in the South (44). This sacred space is often an environment to socialize with one another while engaging in beloved activities such as sewing and playing checkers. Adding methodologies like these are important in public health but first require faculty, staff, and community buy-in as well as strategic planning to explore these methods (21). Meeting interlocutors (i.e., community collaborators) in their intimate settings requires a deep sense of trust that we feel can only be constituted through demonstrations of care and genuine empathy (26).

Collaborative art forms such as photo voice, filmmaking, and music production are gaining traction in health promoting interventions, but the value of these engagement methods also extends to data collection (26, 27). Imagery produced by community collaborators not only disrupts power dynamics in traditional research-participant relationships but also facilitates critical dialog driven by interlocutors (28). Collaborative filmmaking, for example, disrupts power dynamics in research by putting interlocutors in control of what they consider data and what they feel is most valuable for people to know (29). In addition to imagery production, music has also been used to increase visibility of exceptionally vulnerable communities (30). A recent scoping review by Garry et al. (30) found that music is an effective, culturally responsive tool for recruitment and engagement with displaced migrants such as asylum seekers and migrants with precarious status. Arts-based methods show great potential for engaging in trauma responsive data collection and intervention development for culturally diverse communities of interest with increased vulnerability (26).

Literature and storytelling artifacts are also methods for trauma responsive collaboration (31, 32). Historically, art and literature have been utilized to express sentiments that could result in unsafe conditions like political violence (i.e., disappearing, incarceration, execution) (27, 31, 32). Genres like historical fiction, science fiction, and fantasy, for example, are creative spaces for exceptionally vulnerable people to express themselves more freely (31, 32). Some communities may produce cultural artifacts in the form of embroidery, weaving, yarning, and repurposed materials instead of literature but also depict lived experience (33, 34). Embroidery, weaving, yarning, and *cartonera* creation can be valuable ways to engage with vulnerable populations, if granted access to such sacred settings (33, 34). By engaging with creative forms of storytelling produced by our communities of interest, we can begin to understand their historical, political, and social contexts that shape present-day health disparities (20, 26). In our experience, public health students and professionals rarely, if ever, consider the value of fiction literature.

### Classroom and pedagogy

In the classroom, liberatory pedagogies must replace the banking model of education that remains commonplace across academic disciplines (35, 36). Students, especially first generation, come into public health classrooms with relevant personal experiences and will feel more motivated to produce high quality work when given opportunities to connect with the material (35). In our experience, public health faculty often pressure students to distance themselves from their work. As young students, we, the authors, were repeatedly told to dismiss our identities to be good researchers and not bias our results. We view this as a process of dehumanization—both of students and of public health practice—which cuts off the pathway toward community care. In many of our public health courses, we were encouraged to use our positions in the community to gain entrance for research activities but shed our intimate connections with our research subjects to protect the quality of data. This forced displacement of our identities felt like a rejection of our humanity that was to be replaced with a mechanical professionalism. Cultivating student learners to apply their lived experiences to the skills that they learn in school is crucial in generating culturally responsive engagement and innovation (35, 37).

Mentorship from faculty who can relate to underrepresented students from historically excluded and abused communities should be a priority in public health programs (38). More and more marginalized students are entering the academy, but diverse faculty are not (38). Students need mentors who are willing to nurture their interests and who can help identify funding sources that are not as constraining to their project (7, 38). At the very least, public health programs need to grow a faculty base of people who are willing to regularly confront their biases and expand their cultural humility (37, 38).

Revisiting McCroskey and Teven’s (14) foundational study in educational theory and practice, students perform better in classrooms where they feel the instructor genuinely cares for them. Classroom potential for transformative learning occurs when instructors talk less and students work more (39, 40). Guided readings with open prompts such as “What are the main points?” and “How does this information align with what you know?” are easy first steps in facilitating critical dialog while also forging space for considering lived experience (39, 40).

Flores and Román Alfaro (41) suggest a more radical framework of love and care as critical pedagogy. Instructors who create space for students to listen and express traumatic experiences through facilitated conversation cultivate a culture of solidarity in public health leadership (41, 42). Implementation strategies of radical love and care are unique to each instructor, but the main point is to embrace hard conversations and controversial topics (41). Students want and need a space to process their feelings and make sense of their experiences in relation to others around them (42).

On a larger scale, we recommend an intensive examination of approved courses for majors, minors, graduate certificates, electives. How many of these courses extend to Humanities, Social Sciences, and other fields in Liberal Arts? How many approved courses expose students to social determinants from a non-health lens? Opening the parameters of accepted coursework facilitates deeper critical thinking by allowing students the opportunity to connect outside frameworks and theories to health instead of merely regurgitating superficial statements like “everything is public health” (10, 12).

## Conclusion

We have suggested several strategies to reconsider how the public health field can forge stronger, more compassionate leaders. We have explained how the classroom is a critical site for cultivating innovation and collaboration among emerging scholars. We have also provided starting points for community-led collaborative research. Our recommendations are informed by existing literature as well as our personal academic experiences as public health students from communities frequently targeted by public health interventions. Our insights, however, are not exhaustive. It is our intention that the work we have put forth provides a foundation from which the profession can examine internal practices that are stifling growth in leadership development.

## Data Availability

The original contributions presented in the study are included in the article/supplementary material, further inquiries can be directed to the corresponding author.
